# Factors Associated With Sexual Function in Iranian Women With Type 2 Diabetes Mellitus: Partner Relationship as the Most Important Predictor

**DOI:** 10.5812/ircmj.14941

**Published:** 2014-03-05

**Authors:** Zhaleh Shadman, Mahdieh Akhoundan, Nooshin Poorsoltan, Bagher Larijani, Seyed Masoud Arzaghi, Mohsen Khoshniat

**Affiliations:** 1 Endocrinology and Metabolism Research Center, Tehran University of Medical Sciences, Tehran, IR Iran

**Keywords:** Diabetes Mellitus, Hormones, Blood Pressure, Psychology

## Abstract

**Background:**

No comprehensive study has been conducted on risk factors of sexual dysfunction in women with diabetes mellitus.

**Objectives:**

The aim of this study was to consider all possible influencing variables including hormonal, physical and, psychological status, socioeconomic status, and dietary intake to get more accurate and reliable results.

**Patients and Methods:**

Sexual function was assessed by Iranian validated female sexual function index (FSFI).The variables of the study were demographic and diabetes-related factors, stress-depression, physical activity, blood pressure, anthropometric measurements, lipid profile, cortisol, sex and thyroid hormones, 25-hydroxy vitamin D, and dietary intake.

**Results:**

Among all investigated variables, partner relationship showed a strong positive association with FSFI (β = 1.93 ± 0.41, P < 0.0001). In addition, not considering partner relationship, FSFI showed a significant negative association with age (β = -0.19 ± 0.20, P = 0.04), stress-depression score (β = -0.08 ± 0.04, P = 0.04), DD (β = -0.03 ± 0.01, P = 0.04), and systolic blood pressure (β = -0.14 ± 0.06, P = 0.03). Significant associations between FSFI and serum sex hormones and other biochemical were found in neither postmenopausal nor non-menopausal women. The means of SFSI in postmenopausal women were greater than non-menopausal (P = 0.02).

**Conclusions:**

It seems that in our population, female sexual function was much more than just a hormonal or physical problem and psychological factors, especially partner relationship and stress-depression, are the most determinants. In addition, age, duration of challenging with disease, and the lack of controlling systolic blood pressure were common factors that decreased sexual function.

## 1. Background

Sexuality is deepest and innermost feelings of human being. Although sexuality is instinctive, sexual attitudes and behaviors are acquired. Therefore, similar sexual activities might have different meanings for different individuals and from time to time, it might change for an individual. Despite the considerable role of sexual function (SF) as an important determinant of health and wellbeing (1), women SF and its related factors has just recently received some attention (2). However, in spite of high prevalence of sexual dysfunction (SD) (3-5) and studies investigated to reveal the factors associated to female sexual function (FSF) (3-16), it still remains unclear. Although the interference of various diseases and disorders with SD have been investigated (17-21) and diabetes mellitus (DM) as a chronic disease with serious complications is seen with a higher prevalence of SD (2, 13, 17, 20, 22), the cause is not known exactly (13, 23).

## 2. Objectives

In this study, we tried to consider all possible influencing variables including hormonal, physical and psychological status, socioeconomic status, and dietary intake to get more accurate and reliable results.

## 3. Patients and Methods

The Endocrinology and Metabolism Research Center Ethic Committee (EC-00170) approved this cross-sectional study. This cross-sectional study was conducted since 2010 through 2011 with convenience sampling including 420 Iranian women with type 2 diabetes mellitus (T2DM) and mean age of 54.4 ± 9.8 years who lived in Tehran and had been followed for at least two years in Diabetes and Metabolic disease Clinic of Tehran University of Medical. Patients were recruited according to inclusion and exclusion criteria. Inclusion criteria were the age of 35 to 65 years, diagnosis of DM after age of 30 years, having DM for more than five years, and being married. Exclusion criteria were insulin therapy, myocardial infarction, angina pectoralis, stroke, acute liver or renal disease during the past year, chronic inflammation, thyroid disease, genital diseases, vegetarianism, alcohol consumption, smoking, and pregnancy. At the beginning, the protocol and the aim of the study were fully explained to the participants and written informed consent was obtained from each volunteer. Of the approximately 4682 women with DM attending the clinic during the eight months period, 451 eligible women were recognized through patients records and interviews; finally, 428 patients volunteered to participate in the study. The necessary sample size for a similar study was calculated to be 331 women. Based on the assumption that 43% of the female population experiences SD, with an accepted difference of 5% between the sample and the general population and type I error (α) of 0.05 (5), power value of %90 was calculated.

### 3.1. Sexual Function

Patients SF was assessed by the validated 19-item female sexual function index questionnaire (FSFI), a multidimensional self-report instrument for the assessment of FSF in six domains of SF including sexual desire, arousal, lubrication, orgasm, satisfaction, and pain (24, 25). FSFI has an overall reliability of 0.79 to 0.86 for each of the individual domains, a high degree of internal consistency (Cronbach's alpha values of 0.82, 0.70 and higher; P ≤ 0.001), and good construct validity (P < 0.05) in foreign and domestic studies (24, 26). Participants were allowed to complete the FSFI alone in a private room. The questions concern sexual feelings and responses during the past four weeks. The individual domain scores and full-scale (overall) score of the FSFI are derived from the computational formula. For the individual domain scores, the scores of the individual items that comprise the domain are added and multiplied the sum by the domain factor. Total score obtained from the sum of six domain scores ranges from 1.2 to 36. Within the individual domains, a domain score of zero indicates that the subject have had no sexual activity during the past month and a total score of ≤ 26.55 is classified as FSD (27).

### 3.2. Dietary Data and Dietary Pattern

A validated 168-item food frequency questionnaire (FFQ) (28) was completed by trained dietitians through face-to-face interviews to assess the usual dietary intakes of the participants. To estimate portion sizes, a set of two-dimensional shapes and in some cases, three-dimensional food models were used. Amounts were documented in household units, e.g. teaspoons, cups, and grams. Data were analyzed for total calorie intake, carbohydrate, protein, and saturated and unsaturated fatty acids using adjusted N4 software (nutritionist: version 4.0; Tinuviel software, Warrington, UK). To identify dietary patterns, the 168 food items were categorized into 44 food groups based on their similarity of nutrient content and previous Iranian studies (29). However, in some cases that the glycemic loads of foods within a group were a large range; these foods were categorized into separated groups. The reported frequency for each food item was then converted to weekly serving intake.

### 3.3. Physical Activity, Stress-depression and Relationship Questionnaire

Physical activity level (PAL) was assessed by a validated questionnaire in which nine different metabolic equivalent (MET) levels were ranged on a scale from sleep/rest (0.9 METs) to high-intensity physical activities (> 6 METs) (30). For each activity level, the MET value was multiplied by the time spent at that particular level over a 24-hour period. The sum of MET-time at each level and finally, its average was calculated through dividing by 24. Measurement of the three related negative emotional states of depression, anxiety, and tension/stress was done by the self-report validated depression anxiety stress scales (DASS-42) (31). The quality of partner relationship (PR) was scored by ten-item partner relationship questionnaire (PRQ) (32).

### 3.4. Anthropometric Measurements and Blood Pressure

Height was measured with a wall-mounted stadiometer to the nearest 0.1 cm. Weight was determined to the nearest 0.1 kg on the same properly calibrated electronic digital scale (Seca), without shoes, with minimal clothing, and after voiding. Two measurements were obtained and averaged; a third measurement was taken if the first two differed by 0.1. Body mass index (BMI) was estimated as the ratio of body weight to height squared and expressed as kg/m^2^. Waist circumference was determined by placing a measuring tape in a horizontal plane around the abdomen just above the right iliac crest. Two measurements were made to the nearest 0.1 cm and averaged. Both systolic and diastolic blood pressure (SBP and DBP) measuring were done to the nearest 2 mmHg, after resting for at least 15 minutes and sitting on the seat handle. Right arm blood pressure was measured twice with at least five minutes interval by a trained observer using the mercury sphygmomanometer, by Korotkoff's auscultatory method; a third measurement was taken if the first two differed by 2 mmHg.

### 3.5. Biochemical Tests

Ten milliliter, 12-hour fasting state brachial vein blood samples were taken by an expert nurse. Blood sampling in premenopausal women were done at the middle of the menstrual cycle to make the concentration of sex hormones comparable. One milliliter whole blood sample was poured into EDTA containing tubes for the measurement of glycosylated hemoglobin (HbA1c) and the rest of samples were centrifuged at ×3000g for ten minutes at 4˚C, and promptly, serum was transferred into separate tubes that were stored at -80˚C until analyzed. Serum glucose concentration was measured by fluorometry according to glucose oxidase principle (glucose determination kit, Parsazmun, Tehran, Iran) through auto-analyzer instrument (Hitachi 902, Roche, Basel, Switzerland). Glycosylated hemoglobin was determined on whole blood sample by HbA1c Pink Kit and DS5 analyzer. The intra-assay coefficient of variation (CV %) for glucose and HbA1c were 1.4% and 3.7%, respectively, and the inter-assay coefficient of variation were 1.9% and 3.5%, respectively. Serum triglyceride (TG), total cholesterol (TC), LDL (low density lipoprotein), and HDL (high density lipoprotein) cholesterol were measured by the related biochemical kits (Parsazmun, Tehran, Iran) by the auto-analyzer (Hitachi 902, Roche, Basel, Switzerland). The intra-assay CV% were 4.1, 1.3, 2.0, and 1.8, respectively, and the inter-assay CV% were 4.5, 2.0, 2.3, and 2.0, respectively. Serum 25-hydroxy vitamin D was measured by Enzyme-linked immunosorbent assay (ELAISA) (IDS, Boldon, UK). The intra-assay inter-assay CV% were 5.4% and 5.5%, respectively. Serum cortisol, total and free testosterone, estradiol, prolactin, Follicle-stimulating hormone (FSH), Luteinizing hormone (LH), Thyroid-stimulating hormone (TSH), thyroxine (T4), and Triiodothyronine (T3) were measured by enzyme linked immuno assay (ELISA) kit (Monobind, California, USA). The intra-assay coefficient of variation (CV%) were 5.3%, 9.3%, 8.1%, 8.2%, 6.6%, 6.1%, 3.1%, 2.8%, 2.7%, and 1.6% respectively, and the inter-assay coefficient of variation were 5.5%, 10.1%, 8.8%, 7.0%, 6.5%, 4.2%, 3.6%, 3.1%, and 1.9%, respectively.

### 3.6. Statistical Analysis

Data were analyzed with Statistical Package Software for Social Science, version 16 (SPSS Inc., Chicago, IL, USA). Regressions residual plots showed eight outliers that after excluding them the normality of all variables except estradiol, FSH, and LH (distribution of these variables was normal in each group of menopausal and non-menopausal women) were confirmed by Kolmogorov-Smirnov test (P > 0.05). We used independent t test to compare SF between menopausal and non-menopausal women. To determine associated factors to FSFI, at first, we used a simple linear regression for all individual variables. Then, three multivariate linear regression models (including age, stress-depression score, DD, SBP, DBP, HbA1c, FBS, height, waist circumference, BMI, PAL, serum concentrations of TG, TC, LDL, HDL, 25-hydroxy vitamin D3, cortisol, FSH, LH, prolactin, free and total testosterone, estradiol, TSH, T3, and T4) were used to distinguish variables that might affect SF. Another two regression models were used to assess the association of SF with distinguished related variables along with the quality of PR, marital duration (MD), sex frequency in month, number of children, educational status, husband educational status, family income, and women working outside. Pearson correlation was used to assess the association of FSFI with food items, food groups, calorie intake, and proportion of dietary macronutrients. Then adjusted regression was executed. Adjusted estimates of the FSFI mean within the quartiles of percentage of dietary carbohydrate, fiber, protein, total fat, and saturated, mono and poly-unsaturated fats were done. Dietary patterns were derived using Principal Component Analysis (PCA) based on the 168 food items. Sampling adequacy and inter-correlation of variables were supported by KMO (Kaiser-Meyer-Olkin) value of 0.69 and Bartlett’s test of sphericity < 0.0001, respectively. Scree plot was assessed to determine the number of factors, and Varimax rotation was applied to review the correlations between variables and factors. Post-rotated factor loadings showed two dietary patterns and these patterns were labeled based on each food group having the highest loading on each pattern. Food groups with positive loadings in each pattern indicate the direct relationship and food groups with negative loadings show the inverse relationship with that pattern. The factor score for each pattern was calculated by summing the consumption of each food group that were weighted by factor loading and each person received an individual factor score for each identified pattern (33). Then, factor scores were categorized into four groups based on the quartiles of factor score. Linear regression models were used to assess the association of adherence to two major dietary patterns with the mean concentrations of FSFI and concentration of serum hormones in quartiles. In addition, multivariate linear regression model including the age, DD, SBP, and PR was used to distinguish the possible effect of adherence to dietary patterns and other related factors on FSFI. An alpha level of less than 0.05 was accepted in all tests as statistically significant and with the sample size of 420; a power value of 90% was generated.

## 4. Results

Table 1 shows the basic characteristic of patients. In this study, average score of FSFI was estimated 14.61 (minimum: 1.2 and maximum: 31.20). The mean scores in 25, 50, and 75 percentiles was 9, 15.2, and 19.2, respectively. According to FSFI definition, approximately 94.4% of study population suffered from sexual dysfunction. No significant differences were shown in SF score and its components among participated from different ethnic groups. In response to the close-ended question “Do you like sexual relationship in general or not?” 41.5% responded positively. Approximately 79% of patients declared that they have never offered sex to their partners. In response to two open-ended questions “Which characteristic of your spouse increase your sexual affinity?”, 91.9% mentioned kindness, affection, good temper, and being cared for; 4.3%, 1.7%, and 1.7% mentioned husband's hotness, cleanliness, and foreplay as the major points, respectively. In response to “Which characteristic of your spouse decrease your sexual affinity?” %66.6 of respondent named items such as upsetting by husband and low relationship, %22.1 mentioned smell of sweat and smoke, %7 named husband’s low sexual affinity, %3.5 named husband fatigue, and %1.7 mentioned significant age disparity. Sixty-two percent of women with DM stated that DM could affect their SF. Sexual dysfunction was diagnosed in 247 (90.5%) of non-menopausal and 153 (98.7%) menopausal women. Comparison of SF and its components in menopausal and non-menopausal women showed a statistically significant difference between two groups that might not be clinically significant. In addition, in each group, no associations were seen between FSFI and sex hormones. Primary analysis of regression for each variable that might be related to FSFI showed a significant association with age (β= -0.20 ± 0.06, p 0.003), DD (β= -0.01 ± 0.006, P < 0.02), stress-depression (β= -0.07 ± 0.02, P < 0.0001), educational status (β= 0.96 ± 0.44, P < 0.03), number of children (β= -0.80 ± 0.33, P < 0.01), MD (β= -0.14 ± 0.04, P < 0.004), and SBS (β= -0.07 ± 0.02, P < 0.01). Among all investigated variables in three regression models, SF has shown a significant negative association with patients’ age, stress, DD, and SBP (Table 2). Significant association was found between serum sex hormones and other biochemical with SF in neither postmenopausal nor non-menopausal women. Data are not shown.

**Table 1.** Basic Characteristic of Patients ^a^

Variables	Mean ± SD	Percentiles ^b^		
		25	50	75
**Age, y**	54 ± 9	49	55	69
**BMI, kg/m^2^**	29.4 ± 5.1	25.7	28.9	32.1
**DD, y**	10.3 ± 7.7	4	10	15
**WC, cm**	95.6 ± 12.2	88	96	104
**SBP, mmHg**	127 ± 16	120	120	140
**DBP, mmHg**	81 ± 8	80	80	89
**HbA1c, %**	8.2 ± 2.0	6.7	7.9	9.2
**FBS, mg/dL**	159 ± 57	120	150	202
**Triglyceride, mg/dL**	163 ± 82	112	161	209
**Total cholesterol, mg/dL**	170 ± 41	141	162	191
**LDL, mg/dL**	90 ± 24	72	88	107
**HDL, mg/dL**	50 ± 17	45	51	61
**Cortisol, µg/dL**	12.9 ± 5.2	9.0	12.3	16.3
**FSH, mlU/mL**	51.5 ± 34.1	11.2	48.8	82.5
**LH, mlU/mL**	20.5 ± 12.2	9.1	19.0	28.1
**Estradiol, pg/mL**	52.0 ± 74.3	13.1	25.3	60.5
**Prolactin, ng/mL**	8.0 ± 4.2	5.3	7.6	11.9
**Total testosterone, ng/mL**	0.58 ± 0.24	0.40	0.50	0.70
**Free testosterone, Pg/mL**	1.4 ± 0.7	0.9	1.4	1.7
**TSH, µIU/mL**	1.7 ± 1.7	0.80	1.60	2.77
**T4, µg/dL**	8.2 ± 2.3	6.7	7.9	9.4
**T3, ng/mL**	108.1 ± 34.3	85.8	100.1	127.1
**25-OH vitamin D3, nmol/L**	49 ± 30	25	48	71

^a^ Abbreviations: BMI, body mass index; DD, duration of diabetes; DBP, diastolic blood pressure; HbA1c, hemoglobin A1c; FBS, fasting blood sugar; HDL high density lipoprotein, FSH Follicle-stimulating hormone, LDL, low density lipoprotein; LH Luteinizing hormone, SBP, systolic blood pressure; TSH, thyroid-stimulating hormone; T4, thyroxine; T3, triiodothyronine; WC, waist circumference; 25-OH vitamin D3, 25-hydroxy vitamin D3.

^b^ Percentiles of each variable

**Table 2.** Variables Correlate of Sexual Function ^a^, ^b^

	Model 1	P Value	Model 2	P Value	Model 3	P Value
**Subjects in model, No.**	420		413		401	
**Model R ** ^**c**^	0.19		0.25		0.46	
**Variables in model ** ^**c**^						
**Age**	-0.21 ± 0.08	0.011	-0.25 ± 0.09	0.007	-0.19 ± 0.20	0.042
**Stress-depression**	-0.07 ± 0.02	0.002	-0.09 ± 0.02	0.001	-0.08 ± 0.04	0.042
**DD**	-0.01 ± 0.008	0.021	-0.01 ± 0.009	0.041	-0.03 ± 0.01	0.042
**SBP **	-0.10 ± 0.03	0.009	-0.12 ± 0.04	0.007	-0.14 ± 0.06	0.031
**DBP**	0.14 ± 0.09	0.114	0.16 ± 0.09	0.102	-0.01 ± 0.14	0.942
**HbA1C **	0.30 ± 0.35	0.393	0.34 ± 0.53	0.523	1.55 ± 0.84	0.071
**FBS**	-	-	-< 0.0001 ± 0.01	0.972	-0.01 ± 0.01	0.383
**Height**	-	-	16.55 ± 66.42	0.801	122 ± 10	0.251
**WC**	-	-	0.05 ± 0.098	0.581	-0.24 ± 0.15	0.111
**BMI**			0.74 ± 1.63	0.641	3.20 ± 2.56	0.211
**PAL**	-	-	-3.14 ± 3.14	0.324	4.64 ± 4.37	0.293
**Triglyceride **	-	-	-	-	< 0.0001 ± 0.01	0.971
**Total cholesterol**	-	-	-	-	-0.02 ± 0.11	0.842
**HDL**	-	-	-	-	0.01 ± 0.01	0.432
**LDL**	-	-	-	-	0.03 ± 0.14	0.793
**25 (OH) vitamin D**	-	-	-	-	0.007 ± 0.01	0.583
**Cortisol**	-	-	-	-	0.09 ± 0.20	0.638
**FSH**	-	-	-	-	0.07 ± 0.07	0.341
**LH**	-	-	-	-	-0.32 ± 0.19	0.102
**Prolactin**	-	-	-	-	-0.05 ± 0.07	0.519
**Total Testosterone**	-	-	-	-	8.28 ± 7.41	0.258
**Free Testosterone**	-	-	-	-	-1.30 ± 2.39	0.591
**Estradiol**	-	-	-	-	< 0.0001 ± 0.01	0.991
**TSH**	-	-	-	-	-0.12 ± 0.26	0.631
**T3**	-	-	-	-	-0.09 ± 0.49	0.841
**T4**	-	-	-	-	-0.03 ± 0.02	0.221

^a^ All data are presented in Mean ± SD.

^b^ Multivariate linear regression models.

^c^ Parameter estimate.

Results of regression models adding socioeconomic factors are shown in Table 3. Stress is an intermediate between SF and PR; hence, it has not been entered in these regression models. However, after adjusting for PR, the association between stress and FSFI disappeared. In addition, considering PR, the observed associations between FSFI and age, DD, and SBS were not significant anymore (Table 3). Pearson correlation showed significant negative association between PR and anthropometric, biochemical, hormonal, and stress-depression status. Moreover, PR correlated with each FSFI component. No differences were seen in FSFI between those who mentioned tubectomy-hysterectomy history and other patients. Data are not shown.

**Table 3.** Socioeconomic Correlate of Sexual Function ^a^, ^b^, ^c^

	Model 1	P Value	Model 2	P Value	Model 3	P Value
**Subjects in model, No.**	420		420		420	
**Model R ** ^**d**^	0.29		0.34		0.33	
**Variables in model ** ^**d**^						
**MD**	-0.08 ± 0.04	0.027	-0.03 ± 0.05	0.501	0.05 ± 0.07	0.439
**Sex frequency**	0.29 ± 0.45	0.511	0.14± 0.46	0.751	-0.37 ± 0.51	0.471
**PR**	2.02 ± 0.37	< 0.0001	2.12 ± 0.39	< 0.0001	1.93 ± 0.41	< 0.0001
**Number of children**			-0.59 ± 0.43	0.172	-0.52 ± 0.45	0.249
**Educational status**			0.34 ± 0.70	0.623	-0.19 ± 0.74	0.788
**Partner educational status**			-0.23 ± 0.58	0.693	0.04 ± 0.60	0.941
**Working outside**			1.20 ± 1.48	0.422	0.66 ± 1.69	0.691
**Family income**			< -0.0001 ± 0.000	0.722	< 0.0001 ± 0.00	0.681
**Age**	-	-	-	-	-0.17 ± 0.09	0.071
**DD**	-	-	-	-	-0.01 ± 0.006	0.102
**SBP**	-	-	-	-	-0.05 ± 0.02	0.051

^a^ Abbreviations: PR, partner relationship.

^b^ All data are presented in Mean ± SD.

^c^ Multivariate linear regression models.

^d^ Parameter estimate.

Among FFQ food items, Pearson correlation showed significant association between caffeine intake (minimum and maximum intake, 0 and 21 cup/week, respectively with the average intake of 0.5) and FSFI (r = 0.16, P = 0.03). This association was only significant for arousal component of FSFI (r = 0.19, P = 0.01). However, after adjusting for age, PR and SBP, this significance disappeared. No association was found between calorie intake (Kcal/Kg), dietary carbohydrate, protein, and total, saturated, or any kinds of unsaturated fats with FSFI. A negative significant association was detected between percent of dietary protein and orgasm (r = -0.15, P = 0.04). The significance was maintained after adjusting for confounders in regression model (B ± SE: -0.11 ± 0.04, P = 0.02, 95% CI: -0.20- -0.01). Age, SBP, and PR adjusted estimates of the mean concentration of FSFI within the quintiles of percentage of dietary carbohydrate, fiber, protein, total fat, saturated, and mono or poly-unsaturated fats showed no association between SF and dietary macronutrients or calorie intake (data are not shown). Factor analysis revealed two main dietary patterns and the factor loadings for each dietary pattern are presented in Table 4. Food groups with absolute factor loadings >0.20 were considered as having significant contribution to the pattern. These two dietary patterns explained 19.50% of the total variance in food intake. The first pattern with high loadings for Sugar and sweet, ice cream, sweet snacks, seeds, fast foods, soft drinks, mayonnaise, red meats, fried potato, boiled potato, butter and cream, sweeteners, refined grains, salty snacks, salty vegetables, coffee, visceral meat, jelly, and canned fish was labeled “Unhealthy“ dietary pattern. The second pattern, which loaded heavily on vegetables, fruits, nuts, fish, poultry, carrot, banana, liquid oils, low fat dairies, whole grain biscuit, olive, garlic and onion, curd (Cashk), dried fruits, corn, water, and high fat dairies was named “Healthy” dietary pattern. Estimates of the mean concentration of serum cortisol, thyroid, and sex hormones within the quartiles of two dietary patterns are presented in Table 5. Adherence to the "unhealthy" dietary pattern was only associated with serum total testosterone (B = 0.03, P = 0.03, CI: 0.002-0.07). No associations were seen between "healthy" dietary pattern and measured serum hormones. Age, DD, SBP, and PR. Adjusted multivariate linear regression showed no significant association concerning adherence to the two major dietary patterns and FSFI (Table 5). 

**Table 4.** Factor Loading Matrix of Food Groups for Healthy and Unhealthy Dietary Patterns

Food Group	Unhealthy	Healthy
**Sugar and sweet**	0.622	
**Ice cream**	0.547	
**Sweet snacks**	0.487	
**Seeds**	0.472	
**Fast foods**	0.463	
**Soft drinks**	0.450	
**mayonnaise**	0.436	
**Red meats**	0.410	
**Fried potato**	0.402	
**Boiled potato**	0.390	
**Butter and cream**	0.370	-.233
**Sweeteners**	0.367	
**Refined grains**	0.356	
**Salty snacks**	0.339	
**Salty vegetables**	0.312	
**Wholegrain**	-0.286	
**Coffee**	0.271	
**Visceral meat**	0.270	
**Jelly**	0.228	
**Canned fish**	0.209	
**Vegetables**		0.625
**Fruits**		0.593
**Nuts**		0.534
**Fish and poultry**		0.420
**Carrot**		0.418
**Banana**		0.376
**Liquid oils**		0.348
**Low fat dairies**	-0.223	0.344
**Whole grain biscuit**		0.343
**Olive**		0.323
**Garlic and onion**		0.284
**Curd (cashk)**		0.277
**Dried fruits**		0.252
**Corn**		0.237
**Water**		0.237
**High fat dairies**		0.219
**Total variance**	12.37%	7.12%

**Table 5.** FSFI and Serum Hormonal Characteristics of Participants According to Quartiles of Dietary Patterns ^a^

Variable	Quartiles of "Unhealthy" Dietary Pattern				Quartiles of "Healthy" Dietary Pattern			
	Q1	Q2	Q3	Q4	Q1	Q2	Q3	Q4
**Cortisol, µg/dL**	13.4 ± 0.8	12.8 ± 0.5	12.7 ± 0.8	12.7 ± 0.8	13.3 ± 0.6	13.0 ± 0.8	12.0 ± 0.6	13.2 ± 0.8
**FSH, mlU/mL**	50.3 ± 4.8	46.3 ± 5.0	60.2 ± 5.6	48.8 ± 5.2	51.0 ± 5.8	43.0 ± 5.4	58.3 ± 4.8	53.9 ± 4.8
**LH, mlU/mL**	22.8 ± 3.0	18.8 ± 1.9	24.9 ± 2.5	18.1 ± 1.6	22.0 ± 2.6	21.6 ± 3.1	21.1 ± 1.9	20.5 ± 1.7
**Estradiol, pg/ml**	47.1 ± 13.8	54.5 ± 9.1	44.0 ± 6.9	67.5 ± 16.6	42.7 ± 8.3	76.6 ± 15.9	34.2 ± 6.0	55.5 ± 11.9
**Prolactin, ng/mL**	9.3 ± 0.7	12.7 ± 2.2	8.2 ± 0.6	9.1 ± 1.1	10.3 ± 1.2	10.6 ± 1.1	7.3 ± 0.5	11.3 ± 2.0
**Total testosterone, ng/mL**	0.5 ± 0.03	0.5 ± 0.03	0.5 ± 0.02	.7 ± 0.05	0.5 ± 0.02	0.5 ± 0.04	0.5 ± 0.03	0.6 ± 0.04
**Free testosterone, Pg/mL**	1.4 ± 0.1	1.3 ± 0.09	1.2 ± 0.09	1.8 ± 0.1	1.4 ± 0.09	1.5 ± 0.1	1.2 ± 0.09	1.6 ± 0.1
**TSH, µIU/mL**	1.4 ± 0.1	2.5 ± 0.2	1.8 ± 0.1	2.5 ± 0.9	1.8 ± 0.2	2.0 ± 0.1	1.9 ± 0.2	2.4 ± 0.7
**T4, µg/dL**	8.6 ± 0.3	8.0 ± 0.3	8.6 ± 0.2	7.7 ± 0.3	8.4 ± 0.3	8.1 ± 0.3	8.3 ± 0.2	8.2 ± 0.3
**T3, ng/mL**	106.3 ± 4.8	106.5 ± 4.6	113.7 ± 5.2	105.4 ± 5.8	104.8 ± 5.6	111.2 ± 5.3	115.7 ± 5.03	100.5 ± 4.3
**FSFI**	13.3 ± 1.1	16.0 ± 1.0	15.0 ± 1.0	14.1 ± 0.9	14.2 ± 1.1	14.4 ± 0.9	14.2 ± 0.9	15.4 ± 1.2

^a^ Data are presented as Mean ± SE.

## 5. Discussion

Prevalence of sexual dysfunction in our study (approximately %94.4; %90.5 and %98.7 of non-menopausal and menopausal women, respectively) was higher than that previously reported prevalence (3-5, 16, 34, 35) even in patients with DM (2, 13, 22). Among all investigated variables, SF had a negative significant association with PR and maybe with age, stress, DD, and SBP. No significant association was found between serum sex hormones or other biochemical, anthropometric measurements, and socioeconomic status with SF in postmenopausal or non-menopausal women. The most important factors related to SD in patients with DM were age, DD, and menopause (13, 22, 23) along with a decline in FSFI and all aspects of SF with aging (3-5, 36). In the first step, our study confirmed this association by the regression models not considering partner relationship. However, this association disappeared considering this variable. Data showed that FSFI in menopausal women was not clinically lower than non-menopausal (13.34 vs. 15.77) and no associations was seen with sex hormones. Some studies have reported increase in sexual dysfunction after menopause (4-6). Some others mentioned sex hormones as modifying factor (3, 5, 37, 38). Our results were in agreement with those of previous studies in which hypertension was associated with SD (3, 36). However, considering PR, this association got weaker. It is assumed that mechanism of worsening SD along increasing blood pressure is that the increased blood pressure leads to remodeling of the blood vessel wall and impaired blood supply of peripheral tissues. Diminished genital blood flow secondary to atherosclerosis might result in clitoral and vaginal vascular insufficiency, resulting in vasculogenic FSD (8). Our results were in agreement with those of previous studies in which depression and stress were associated with SD (3, 8, 10, 11, 16). Psychological factors, e.g. anxiety, fatigue, pain, feeling of guilt, anti-masculine feelings, and embarrassment in sexual relationships were reported to be higher in Iranian anorgasmic women (12). There have been a significant positive correlation between women SF and its components and marital adjustment (35). Moreover, PR has been reported as an important factor in determining the quality of Iranian women SF (16). Intimate relations scores were higher in those with normal SF in comparison to those with SD (39). It seems that the association between FSFI and PR is a stronger factor than stress-depression status and some psychological and ideological factors might influence FSF through PR. In some studies, stress induced cortisol was associated with lower SF (40, 41). The participated patients in our study were not affected by diabetes complications and most of them were in the normal range of cortisol levels. Therefore, our study was not able to detect any actual association between FSFI and cortisol. Our results were inconsistent with the previous studies that detected some associations between FSFI and MD (34),educational level (16), husband’s educational level (11, 16), economical status (16), hysterectomy (4), or physical activity (3). We could not definitely rule out the effect of these factors. Emotional and learned responses were shown to be the most determining factors in FSF (37). It seems that the low score for relationship masked the effects of other possible effective factors in present study. Furthermore, 41.5% of patients reported their interest in sex and 79% had never offered sex to their partners, which might reflect a particular educational or socio-cultural background concerning sex. Such that 91.9% mentioned kindnesses, affection, good temper, and being cared for and %66.6 of respondents named items such as upsetting by husband and low relationship as increasing and decreasing affinity to sex, respectively. The past performance and other relevant factors such as the emotional status towards their husband, changes in the status of the partner, and sexual response were more important in comparison to sex hormones in determining of SF (42). Adherence to the "unhealthy" dietary pattern was only associated with serum total testosterone but not with FSFI. Few studies on dietary pattern and FSFI had reported that Mediterranean diet was directly related to the FSF (13, 14). With regard to no association between food items and SF, it should be noticed that our results were applied for food intake of our studied population and did not include less or more amounts. It is possible that some food items in higher amounts could have had some effects on SF, which was not identified in our study. The observed association between coffee intake and FSFI might be related to the effect of enhancing mental energy as sympathomimetic agent (43) that was only significant for arousal component of FSFI. However, after adjusting for age, PR, and SBP, this significance disappeared. In addition, a significant negative association was detected between dietary protein and orgasm that might influence female's orgasm through unknown mechanisms.

The most important strong point of this study was assessing all possibly related factors together as far as possible. The most weakness, due to the high prevalence of sexual dysfunction in the study population;. We could not definitely conclude that other studied factors had no effects on FSF in other physiological conditions. Rather than diabetics sex dysfunction is caused by physiological problems, seems to link to psychological factors. In our study, partner relationship was the most established risk factor for FSF in patients with DM. In addition, after psychological factors, age, duration of challenging with disease, and the lack of controlling SBP were common factors that decrease SF in women with T2DM. Age and DD are not modifiable, Hence, interventions should be focused on improving relationship, reducing psychological problems, and controlling blood pressure.
